# Single-Incision vs. Multiport Robotic Sacrocolpopexy: 126 Consecutive Cases at a Single Institution

**DOI:** 10.3390/jcm10194457

**Published:** 2021-09-28

**Authors:** Gina Nam, Sa-Ra Lee, A-mi Roh, Ju-Hee Kim, Sungwook Choi, Sung-Hoon Kim, Hee-Dong Chae

**Affiliations:** 1 Department of Obstetrics and Gynecology, Chung-Ang University Hospital, Chung-Ang University College of Medicine, 102, Heukseok-ro, Dongjak-gu, Seoul 06973, Korea; ginanam@cau.ac.kr; 2 Asan Medical Center, Department of Obstetrics and Gynecology, University of Ulsan College of Medicine, 88, Olympic-ro 43-gil, Songpa-gu, Seoul 05505, Korea; xjuheex@gmail.com (J.-H.K.); palpitation@hanmail.net (S.C.); kimsung@amc.seoul.kr (S.-H.K.); hdchae@amc.seoul.kr (H.-D.C.); 3 Department of Obstetrics and Gynecology, Wonder Women’s Clinic, 1074, Gyeongui-ro, Paju-si 10908, Gyeonggi-do, Korea; spatialism@gmail.com

**Keywords:** pelvic organ prolapse, recurrence, robot-assisted laparoscopic sacrocolpopexy, single incision

## Abstract

Robot-assisted laparoscopic sacrocolpopexy (RSC) has gained popularity as a method for easier intracorporeal suturing than conventional laparoscopic sacrocolpopexy. However, few studies have compared multiport RSC (MP-RSC) and single-incision RSC (SI-RSC). We aimed to compare perioperative outcomes between these techniques for advanced pelvic organ prolapse (POP). We analyzed 126 patients who underwent RSC for POP quantification (all stage III to IV) between March 2019 and May 2021 at Seoul Asan Medical Center. We prospectively collected operation-related data, including total operation time (OT; from skin incision to closure) and perioperative outcomes. A total of 106 and 20 patients underwent MP-RSC and SI-RSC, respectively. The mean ages were 57.49 ± 10.89 and 56.20 ± 10.30 years in the MP-RSC and SI-RSC groups, respectively. The mean total OT was significantly shorter for MP-RSC than for SI-RSC (105.43 ± 24.03 vs. 121.10 ± 26.28 min). The OT difference was 15.67 min (95% confidence interval, 3.90–25.85, *p* = 0.009). No statistically significant differences were observed in terms of perioperative variables (estimated blood loss, hospital stay) and postoperative adverse events (POP recurrence, mesh erosion). SI-RSC had comparable intraoperative and postoperative outcomes to MP-RSC, with additional cosmetic benefits. MP-RSC had significantly shorter OT than SI-RSC.

## 1. Introduction

Pelvic organ prolapse (POP) is a relatively common condition that is reported to be evident on gynecologic examination in 40% to 60% of parous women [1,2]. The importance of POP is increasing in aging societies owing to its high prevalence of 30% among women aged >50 years [3]. The probability that a woman will undergo surgery for POP by 80 years of age is estimated to be 20% [4]. Historically, abdominal sacrocolpopexy (SC) has been recognized as the gold-standard surgical treatment for apical compartment prolapse. In SC, a mesh is attached to the cervix or vagina and suspended to the anterior longitudinal ligament of the sacrum [5,6]. The procedure has been reported to have a higher success rate (78–100%) than vaginal approaches for apical prolapse [5].

Laparoscopic SC is known to be as effective as abdominal SC, but laparoscopy is associated with reduced blood loss and shorter length of hospital stay. With technical improvements in intracorporeal laparoscopic suturing, laparoscopic SC has become a more popular technique than abdominal SC [7]. The introduction of robotic systems has enabled much easier intracorporeal suturing and deep vesicovaginal and rectovaginal dissections. Therefore, the use of robotic SC continues to increase.

Laparoendoscopic single-port surgery (LESS) has been developed to provide cosmetic benefits via minimal skin incision; however, difficult angulations and collisions between laparoscopic instruments are the main technical challenges limiting its feasibility [8]. In 2014, the da Vinci Single-Site^®^ platform (Intuitive Surgical Inc., Sunnyvale, CA, USA) was developed to overcome the limitations of LESS; however, the weak power transference achievable by semirigid instruments with limited ranges of motion precludes the application of the Single-Site system to diverse types of procedures. More recently, the Single-Port^®^ system (Intuitive Surgical, Sunnyvale, CA, USA) was developed to overcome the technical limitations of Single-Site and the newer system has been used for more indications than the Single-Site platform.

We first reported on the feasibility and safety of single-incision robot-assisted laparoscopic SC (SI-RSC) in 2016 [9]. Since then, a few studies have reported successful surgical outcomes using this technique [10,11,12]. However, only a few studies have compared operative outcomes between multiport robot-assisted laparoscopic SC (MP-RSC) and SI-RSC. Therefore, this study aimed to compare perioperative surgical outcomes, including total operation time (OT) and follow-up outcomes between MP-RSC and SI-RSC.

## 2. Materials and Methods

### 2.1. Study Design and Patients

Among 288 patients who underwent RSC performed by a single surgeon (S.R.L.)—between January 2015 and February 2019 at Ewha Womans University Mokdong Hospital, as well as between March 2019 and May 2021 at Seoul Asan Medical Center—a total of 126 consecutive patients who underwent RSC between March 2019 and May 2021 at Seoul Asan Medical Center were included in this study to minimize the bias associated with intrapersonal surgical proficiency and institution. A retrospective chart review was performed on prospectively collected data.

We obtained the following data from each patient’s medical chart: age; body mass index (BMI); detailed gynecologic, medical, and surgical histories; and POP quantification (POP-Q) stage. Furthermore, we obtained the following surgery-related data: concurrent surgery, anesthesia time, and total OT (defined as the time from skin incision to the completion of skin closure, including all concurrent operations). Additionally, we collected data on perioperative outcomes, including estimated blood loss, any intraoperative or postoperative adverse events, length of hospital stay, postoperative fever within 48 hours, and other surgical complications. Postoperative follow-up data on the recurrence of POP were also collected.

### 2.2. Surgical Procedures

**Figure 1:**
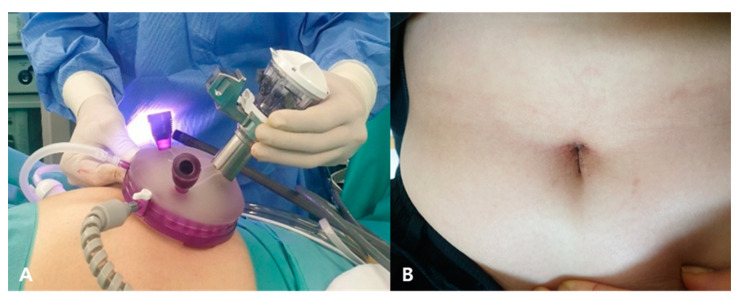
Single 25–27 mm intraumbilical incision was made and a multichannel single port was used in the single-incision robotic approach, Single-Port system in this case (**A**) Intraoperative (**B**) Postoperative 2 weeks follow-up visit.

The surgical steps were the same as described in our previous report [9]. All operations were performed under general anesthesia and all patients underwent standard operative care. SI-RSC included both Single-Site and Single-Port robotic approaches. The da Vinci Si^®^ and da Vinci Xi^®^ systems (Intuitive Surgical, Sunnyvale, CA, USA) with central or side docking were used for MP-RSC and Single-Site SI-RSC. The da Vinci SP^®^ system (Intuitive Surgical, Sunnyvale, CA, USA) with central docking was used for Single-Port SI-RSC. In MP-RSC, a total of three ports (reduced ports) were used. One 25–27 mm intraumbilical incision was made and a multichannel single port was inserted for the camera and the assistant’s laparoscopic instruments. Two 8 mm robotic ports were placed in the flank: one on the left side and one on the right side of the umbilicus, 8–10 cm away from the intraumbilical incision. In SI-RSC, a single 25–27 mm intraumbilical incision was made for multichannel single-port insertion (Figure 1).

The intracorporeal surgical procedures were identical for MP-RSC and SI-RSC. Supracervical hysterectomy with or without adnexectomy was performed and the cervical stump was sutured with 1-0 V-Loc (Covidien, Mansfield, MA, USA) using a continuous running suture technique. Moreover, we used the remaining thread to create traction by grasping the thread during the process of mesh fixation to the anterior and posterior vaginal wall. This step was omitted in patients with vault prolapse. The avascular anterior vesicovaginal plane and posterior rectovaginal plane were dissected by approximately 6–7 cm in length and the mesh was sutured to fix approximately 5–6 cm of the anterior and posterior vaginal wall. The cranial end of the Y-shaped mesh was fixed using nonabsorbable sutures after adjusting the mesh tension. The peritoneum was completely closed with absorbable barbed sutures—to prevent mesh exposure, bowel adhesion and bowel strangulation—using the peritoneal tunneling method [13]. The retrieved uterus and adnexa were removed using knife morcellation within a contained bag.

The same surgical materials, including sutures and mesh, were used for all patients. The mesh used for surgery was a partially absorbable Y-shaped polypropylene mesh (Seratex^®^ PA B2 type; Serag-Wiessner KG, Naila, Germany). We placed 12–15 anchoring sutures on the cervical stump or tip of the vaginal vault (2–3 sutures), anterior and posterior vaginal wall (6–8 sutures each) and presacral anterior longitudinal ligament (3 or 4 sutures) with nonabsorbable 2-0 Prolene (Johnson & Johnson Medical GmbH, Norderstedt, Germany) or absorbable 2-0 PDS (Ethicon, Somerville, NJ, USA).

Complete peritoneal closure was performed using absorbable barbed sutures (2-0 Monofix PDO; Samyang, Daejeon, Korea) or 1-0 Quill™ SRS bidirectional barbed sutures (Angiotech Pharmaceuticals Inc., Vancouver, BC, Canada).

### 2.3. Statistical Analysis

To compare continuous variables between the MP-RSC and SI-RSC groups, we used Student’s *t*-test. To compare the proportions of categorical variables between the two groups, we used Fisher’s exact test. Statistical analysis was performed using SPSS for Windows (version 20; IBM Corp., Armonk, NY, USA).

## 3. Results

### 3.1. Patients’ Baseline Characteristics

We included 126 consecutive patients with advanced POP who underwent RSC during the study period in the analysis, among whom 106 and 20 underwent MP-RSC and SI-RSC, respectively. The baseline characteristics of the patients are summarized in Table 1. The mean patient ages were 57.49 ± 10.89 and 56.20 ± 10.30 years, the mean BMIs were 23.99 ± 3.52 and 22.95 ± 2.44 kg/m^2^ and the median parity was 2 and 2 in the MP-RSC and SI-RSC groups, respectively. Thirty-eight (35.8%) patients in the MP-RSC group and 3 (15%) patients in the SI-RSC group (*p* = 0.07) had undergone previous pelvic surgery, including hysterectomy. Sixteen (15.1%) patients in the MP-RSC group and no patients in the SI-RSC group had vault prolapse or had undergone a hysterectomy (*p* = 0.07). The distribution of POP-Q stage was not significantly different between the two groups. Most patients had POP-Q stage III in both the MP-RSC (85%) and SI-RSC (95%) groups. All patients had accompanying apical compartment prolapse and the second main compartment of prolapse was the anterior compartment (51.9% in the MP-RSC group and 75% in the SI-RSC group).

### 3.2. Comparison of Surgical Outcomes between MP-RSC and SI-RSC

The mean OT was significantly shorter in the MP-RSC group (105.43 ± 24.03 min) than in the SI-RSC group (121.10 ± 26.28 min). The mean anesthesia time was also significantly shorter in the MP-RSC group (Table 2). The mean differences in each surgical times were as follows: OT, 15.67 min (95% confidence interval (CI), 3.90–25.85, *p* = 0.009); anesthesia time, 13.32 min (95% CI, 0.79–27.43, *p* = 0.037). The types and distributions of concurrent procedures were not significantly different between the groups. Among the MP-RSC and SI-RSC procedures, adnexectomy was performed in 68 (64.2%) and 12 (60.0%), adhesiolysis in 12 (11.3%) and 2 (10.0%), posterior colpoperineorrhaphy in 15 (14.2%) and 4 (20.0%), and transobturator tension-free vaginal tape surgery in 34 (32.1%) and 9 (45.0%) patients, respectively. The mean estimated blood loss was lower in the SI-RSC group than in the MP-RSC group (37.00 ± 31.64 vs. 55.24 ± 51.93 mL); however, the difference was not statistically significant (*p* = 0.93). The median length of hospital stay was similar between the two groups (2 days, *p* = 0.93).

In the subgroup of patients who underwent MP-RSC and SI-RSC with concurrent hysterectomy (excluding those who had previously undergone hysterectomy), the mean OT was significantly shorter in the MP-RSC group (Table 3). The median hospitalization durations were not different between the groups.

### 3.3. Comparison of Intraoperative and Postoperative Adverse Surgical Outcomes

Intraoperative and postoperative adverse events are listed in Table 4. The numbers of adverse events did not statistically differ between the groups. Bladder injury occurred in three patients in the MP-RSC group and in one patient in the SI-RSC group; all of these patients underwent intraoperative primary repair with extended urinary drainage with an indwelling Foley catheter for 7 days and had no further complications. Bowel injury did not occur in either group. No significant intergroup differences were observed in terms of postoperative hemoglobin changes, transfusion requirements, postoperative fever, urinary tract infection, urinary retention, constipation, back pain or de novo stress urinary incontinence. One case of retroperitoneal abscess from a postoperative hematoma occurred in the MP-RSC group. As the mesh was not infected, mesh removal was not needed because intravenous antibiotics and abscess drainage resolved the problem. Two cases of umbilical wound infection occurred in the MP-RSC group. One case of vaginal mesh erosion occurred in the MP-RSC group; it involved the high posterior vaginal wall and was observed at the 3-month postoperative follow-up visit. After removing the exposed mesh under local anesthesia, primary vaginal repair was performed. Oral estrogen combined with local vaginal estrogen application for 4 weeks resolved the problem without recurrence after 6 months of follow-up. At the 4-week postoperative follow-up point, there was no recurrent POP in the SI-RSC group. Four patients in the MP-RSC group were diagnosed with POP-Q stage I anterior compartment prolapse after 4 postoperative weeks; however, they had no symptoms and did not need further interventions.

The types and frequencies of intraoperative and postoperative adverse events did not differ significantly between the groups when patients with previous hysterectomies were excluded from the analysis (Table 5). Bladder injury occurred in one patient in the MP-RSC group and one patient in the SI-RSC group. No significant intergroup differences were observed in terms of postoperative hemoglobin changes, transfusion requirements, postoperative fever, urinary tract infection, urinary retention, retroperitoneal abscess, constipation, back pain, wound infection, posterior vaginal wall mesh erosion, de novo stress urinary incontinence or recurrence of POP.

## 4. Discussion

In this retrospective comparative study of SC, we investigated the feasibility and perioperative outcomes of MP-RSC and SI-RSC. SI-RSC showed similar surgical and anatomical outcomes to MP-RSC and both techniques were feasible and safe options for treating apical POP.

The mean OT was shorter among our SI-RSC cases than that associated with laparoscopic SC (272 min, *n* = 273) and abdominal SC (222 min, *n* = 589) in a cohort study comparing minimally invasive SC and abdominal SC [14]. The mean OT among our SI-RSC cases was also shorter than that associated with MP-RSC (275 min, *n* = 121) and laparoscopic SC (235 min, *n* = 249) in another cohort study [15]. However, other studies have reported conflicting results. Paraiso et al. reported that laparoscopic SC was associated with a shorter mean OT than RSC (199 vs. 265 min, *p* < 0.001) and that RSC caused more pain to patients than laparoscopic SC [16]. Pan et al. also compared laparoscopic SC with RSC and found that the mean OT was longer in the RSC group than in the laparoscopic SC group (245.9 vs. 205.9 min, *p* < 0.001) [17].

The mean OTs in our study (105.43 min for MP-RSC and 124.10 min for SI-RSC) were much shorter than those determined by a recent randomized controlled trial (157.5 min for MP-RSC and 181.3 min for SI-RSC, *n* = 32) [18], as well as that determined by a case series on SI-RSC (190 min, *n* = 25) [12]. In 2018, a study by Liu et al. involving 15 SI-RSC cases determined a mean OT of 74.7 min; however, the published report did not specify whether OT meant the console time or the time from skin incision to skin closure [11]. Notably, the mean OTs for MP-RSC and SI-RSC in our study were shorter than those reported in previous publications. Supracervical hysterectomy, rather than total hysterectomy, was performed in our study and this can be a reason for the shorter OTs. Considering that most patients with POP are older than 65 years, decreasing the total OT is important for minimizing perioperative complications. The shorter OTs in our study might also be due to the surgeon’s experience, which included a total of 288 RSC cases, with the last 126 cases performed after the initial learning curve.

As expected, the mean OT in our study was longer (by 15.67 min) for SI-RSC than for MP-RSC. This can be explained by technical difficulties associated with SI-RSC triangulation, semirigid instruments and collision of instruments, although most cases (18 of 20) in the SI-RSC group were performed using the Single-Port platform, which was recently developed to overcome the disadvantages of the Single-Site platform [19].

Over time, the field of minimally invasive surgery has continuously improved, resulting in reduced morbidity and better cosmesis, especially with the evolution of robotic surgical units [20]. Efforts to improve the da Vinci surgical system (Intuitive Surgical) have focused on surgical maneuverability, improvements in ergonomics and reductions in port size and number. The introduction of the Single-Site wristed needle driver to overcome these technical obstacles has enabled procedures that require multiple laparoscopic sutures and knot tying, such as laparoscopic myomectomy and SC [9]. We previously presented the first six SI-RSC cases performed using the Single-Site platform and provided a detailed description of the technique using the Single-Site wristed needle driver [9]. We also reported the first case of Single-Port RSC through a video presentation in 2019 at the annual congress of the American Association of Gynecologic Laparoscopists. Recently, we compared two types of SI-RSC and showed that both Single-Site RSC and Single-Port RSC are feasible and effective surgical options for symptomatic apical POP, with cosmetic benefits [21]. Although we did not perform a learning curve analysis, we strongly suggest that a beginner starts with MP-RSC first and proceeds to SI-RSC after gaining proficiency in MP-RSC. This can shorten the learning curve associated with SI-RSC.

As expected, specimen retrieval was equally easy during both MP-RSC and SI-RSC because we performed the same length of umbilical incision. This was possible because we used a multichannel single port to decrease the number of skin incisions even in MP-RSC (i.e., reduced ports: a total of three ports including the intraumbilical incision). A recently published randomized controlled trial comparing MP-RSC and SI-RSC reported that postoperative incisional herniation occurred in 6.2% of patients in the SI-RSC group compared with 0% in the MP-RSC group [18]. In another study, two cases of incisional hernia were observed in the Single-Site RSC group, both in obese patients [21]. Similarly, in our study, two cases of umbilical wound herniation occurred in obese patients, although these patients underwent MP-RSC. This result confirmed that obesity is a risk factor for incisional hernia development. Therefore, special caution should be taken to prevent incisional herniation when performing RSC in obese patients.

A previous study demonstrated that LESS was associated with significantly reduced postoperative pain, faster recovery, improved cosmesis, lower costs, and reduced morbidity related to multiport surgery [22]. A study on robotic LESS reported high patient satisfaction (based on the Patient and Observer Scar Assessment Scale) in terms of the overall appearance of surgical scars [23]. Cosmetic satisfaction has important functional and psychological implications. Therefore, SI-RSC can be expected to decrease postoperative pain with increased cosmetic satisfaction even for older women.

This study had several strengths. First, this was a comparison study of MP-RSC and SI-RSC based on data from all consecutive operations performed by a single urogynecologist. This single surgeon’s experience, involving recent surgical cases, minimized the influence of variations in surgical proficiency across different surgeons. Second, we used a validated POP quantification system both preoperatively and postoperatively. Third, the surgeon who performed the procedures in this study reported the first cases of both Single-Site and Single-Port RSC worldwide.

However, this study also had some limitations. First, this was a single-center retrospective study and not a multicenter randomized controlled trial. This can limit the study’s generalizability; therefore, to demonstrate the reproducibility of our study findings, we should perform an additional study involving procedures performed by multiple surgeons. Second, our study had a relatively small sample size, with a particularly small SI-RSC group. Third, the relatively short-term follow-up data precluded comparisons of long-term efficacy and safety between MP-RSC and SI-RSC. Finally, we only assessed perioperative outcomes without preoperative and postoperative assessments on quality of life or subjective success.

However, the purpose of this study was to compare the OTs and perioperative outcomes associated with the two techniques. The longer mean OT associated with SI-RSC relative to MP-RSC, especially for the incision, docking, hysterectomy, and suturing components, was due to limitations in free angulation and the relatively weak power transference attainable by semirigid instruments, including the wristed needle driver. Therefore, a longer learning curve can be expected for SI-RSC and more experience is required to gain proficiency at performing SI-RSC.

## 5. Conclusions

In this retrospective comparison study, SI-RSC was associated with a longer mean OT but equivalent perioperative outcomes and better cosmesis than MP-RSC. Therefore, SI-RSC is a feasible and safe surgical option comparable to MP-RSC with additional cosmetic benefits.

## Figures and Tables

**Table 1:** Sociodemographic data and clinical history of patients.

Characteristics	MP-RSC (*n* = 106)	SI-RSC (*n* = 20)	*p*-Value
Age (years), mean ± SD ^a^	57.49 ± 10.89	56.20 ± 10.30	0.63
BMI (kg/m^2^), mean ± SD ^a^	23.99 ± 3.52	22.95 ± 2.44	0.21
Menopause, *n* (%) ^b^	71 (67.0)	11 (55.0)	0.30
Parity, median [range] ^a^	2 [0–9]	2 [1–3]	0.68
Hypertension, *n* (%) ^b^	28 (26.4)	6 (30.0)	0.74
Diabetes, *n* (%) ^c^	6 (5.7)	3(15.0)	0.15
Asthma, *n* (%) ^c^	4 (2.8)	0	1
Constipation, *n* (%) ^c^	5 (4.7)	0	1
Current stress incontinence, *n* (%) ^b^	44 (41.5)	11 (55.0)	0.27
Current overactive bladder, *n* (%) ^c^	9 (8.5)	0	0.35
Previous prolapse repair surgery, *n* (%) ^c^	10 (9.4)	0	0.36
Previous stress incontinence repair surgery, *n* (%) ^c^	7 (6.6)	1 (5.0)	1
Previous pelvic surgery, *n* (%) ^b^	38 (35.8)	3(15.0)	0.07
Vault prolapse, *n* (%) ^c^	16 (15.1)	0	0.07
POP-Q stage, *n* (%) ^b^			0.47
Stage III	91 (85.8)	19 (95.0)	
Stage IV	15 (14.2)	1 (5.0)	
Main compartment of prolapse, *n* (%) ^b^			0.06
Anterior	55 (51.9)	15 (75)	
Apical	51 (48.1)	5 (25.0)	
Second, compartment of prolapse, *n* (%) ^c^			0.16
Anterior/posterior	50 (47.2)/1(0.9)	5 (25.0)/0	
Apical	55 (51.9)	15 (75.0)	

MP-RSC, multiport robot-assisted laparoscopic sacrocolpopexy; SI-RSC, single-incision robot-assisted laparoscopic sacrocolpopexy; BMI, body mass index; POP-Q, pelvic organ prolapse quantification; SD, standard deviation, ^a^ *p*-value was calculated using an independent *t*-test, ^b^ *p*-value was calculated using the chi-square test, ^c^ *p*-value was calculated using Fisher’s exact test.

**Table 2:** Surgical and perioperative data.

	MP-RSC (*n* = 106)	SI-RSC (*n* = 20)	Mean Difference (95% CI)	*p*-Value
Total operation time (min), mean ± SD ^a^	105.43 ± 24.03	121.10 ± 26.28	15.67 (3.90–25.85)	0.009
Anesthesia time (min), mean ± SD ^a^	149.93 ± 25.55	163.25 ± 28.14	13.32 (0.79–27.43)	0.037
Concurrent surgery, *n* (%)				
Adnexectomy ^b^	68 (64.2)	12 (60.0)	-	0.72
Adhesiolysis ^c^	12 (11.3)	2 (10.0)	-	1
Posterior colpoperineorrhaphy ^c^	15 (14.2)	4(20.0)	-	0.50
TOT ^b^	34 (32.1)	9 (45.0)	-	0.26
Estimated blood loss (mL), mean ± SD ^a^	55.24 ± 51.93	37.00 ± 31.64	-	0.13
Length of hospital stay (days), median [range] ^a^	2 [1–5]	2 [1–6]	-	0.93

MP-RSC, multiport robot-assisted laparoscopic sacrocolpopexy; SI-RSC, single-incision robot-assisted laparoscopic sacrocolpopexy; TOT, transobturator tension-free vaginal tape; SD, standard deviation; CI, confidence interval, ^a^ *p*-value was calculated using an independent *t*-test, ^b^ *p*-value was calculated using the chi-square test, ^c^ *p*-value was calculated using Fisher’s exact test.

**Table 3:** Surgical and perioperative data excluding vault prolapse patients.

	MP-RSC (*n* = 90)	SI-RSC (*n* = 20)	Mean Difference (95% CI)	*p*-Value
Total operation time (min), mean ± SD ^a^	107.14 ± 24.75	121.10 ± 26.28	13.96 (1.69–26.22)	0.026
Anesthesia time (min), mean ± SD ^a^	151.42 ± 26.39	163.25 ± 28.14	11.83 (1.25–24.91)	0.076
Concomitant surgery, *n* (%)				
Adnexectomy ^b^	63 (70.0)	12 (60.0)		0.39
Adhesiolysis ^c^	8 (8.9)	2 (10.0)		1.00
Posterior colpoperineorrhaphy ^c^	13 (14.4)	4 (20.0)		0.51
TOT ^b^	30 (33.3)	9 (45.0)		0.32
Estimated blood loss (mL), mean ± SD ^a^	57.78 ± 53.82	37.00 ± 31.64		0.10
Length of hospital stay (days), median [range] ^a^	2 [1–5]	2 [1–6]		0.81

MP-RSC, multiport robot-assisted laparoscopic sacrocolpopexy; SI-RSC, single-incision robot-assisted laparoscopic sacrocolpopexy; TOT, transobturator tension-free vaginal tape; SD, standard deviation; CI, confidence interval, ^a^ *p*-value was calculated using an independent *t*-test, ^b^ *p*-value was calculated using the chi-square test, ^c^ *p*-value was calculated using Fisher’s exact test.

**Table 4:** Comparison of intraoperative and postoperative adverse events.

	MP-RSC (*n* = 106)	SI-RSC (*n* = 20)	*p*-Value
Intraoperative AEs, *n* (%)			
Bladder injury ^b^	3 (2.8)	1 (5.0)	0.50
Bowel injury	0	0	
Postoperative AEs, *n* (%)			
Hb decrease (g/dL), median [range] ^a^	−1.8 [−3.10 to 0.2]	−1.8 [−4.30 to −0.3]	0.98
Packed cell transfusion ^b^	5 (4.7)	2 (10.0)	0.30
Fever ^b^	9 (8.5)	0	0.35
Urinary tract infection ^b^	2 (1.9)	0	1
Urinary retention ^b^	0	1 (5.0)	0.16
Retroperitoneal abscess ^b^	1 (0.9)	0	1
Constipation ^b^	6 (5.7)	0	0.59
Back pain ^b^	1 (0.9)	2 (10.0)	0.07
Wound infection ^b^	2 (1.9)	0	1
Posterior vaginal wall—mesh erosion ^b^	1 (1)	0	1
De novo stress urinary incontinence ^b^	10 (9.4%)	1 (5.0)	1
POP recurrence (stage I) ^b^	4 (3.8)	0	1

MP-RSC, multiport robot-assisted laparoscopic sacrocolpopexy; SI-RSC, single-incision robot-assisted laparoscopic sacrocolpopexy; AEs, adverse events; Hb, hemoglobin; POP, pelvic organ prolapse, ^a^ *p*-value was calculated using an independent *t*-test, ^b^ *p*-value was calculated using Fisher’s exact test.

**Table 5:** Comparison of intraoperative and postoperative adverse events excluding patients with a history of hysterectomy.

	MP-RSC (*n* = 90)	SI-RSC (*n* = 20)	*p*-Value
Intraoperative AEs, *n* (%)			
Bladder injury ^b^	1 (1.1)	1 (5.0)	0.33
Bowel injury	0	0	
Postoperative AEs, *n* (%)			
Hb decrease (g/dL), median [range] ^a^	−1.8 [−3.10 to 0.2]	−1.8 [−4.30 to −0.3]	0.72
Packed cell transfusion ^b^	3 (3.3)	2 (10.0)	0.22
Fever ^b^	9 (10.0)	0	0.21
Urinary tract infection ^b^	1 (1.1)	0	1
Urinary retention ^b^	0	1 (5.0)	0.18
Retroperitoneal abscess ^b^	1 (1.1)	0	1
Constipation ^b^	4 (4.4)	0	1
Back pain ^b^	1 (1.1)	2 (10.0)	0.09
Wound infection ^b^	1 (1.1)	0	1
Posterior vaginal wall—mesh erosion ^b^	1 (1.1)	0	1
De novo stress urinary incontinence ^b^	8 (8.9)	1(5.0)	1
POP recurrence (stage I) ^b^	4 (4.4)	0	1

MP-RSC, multiport robot-assisted laparoscopic sacrocolpopexy; SI-RSC, single-incision robot-assisted laparoscopic sacrocolpopexy; AEs, adverse events; Hb, hemoglobin; POP, pelvic organ prolapse, ^a^ *p*-value was calculated using an independent *t*-test, ^b^ *p*-value was calculated using Fisher’s exact test.

## Data Availability

The Excel (Microsoft Corp., Redmond, WA, USA) data used to support the findings of this study were supplied by S.-R.L. under license and requests for access to these data should be made to S.-R.L.

## References

[1] Handa V.L., Garrett E., Hendrix S., Gold E., Robbins J. (2004). Progression and remission of pelvic organ prolapse: A lon-gitudinal study of menopausal women. Am. J. Obstet. Gynecol..

[2] Hendrix S.L., Clark A., Nygaard I., Aragaki A., Barnabei V., McTiernan A. (2002). Pelvic organ prolapse in the Women’s Health Initiative: Gravity and gravidity. Am. J. Obstet. Gynecol..

[3] Chow D., Rodríguez L.V. (2013). Epidemiology and prevalence of pelvic organ prolapse. Curr. Opin. Urol..

[4] Smith F.J., Holman C.D.J., Moorin R., Tsokos N. (2010). Lifetime risk of undergoing surgery for pelvic organ prolapse. Obstet. Gynecol..

[5] Maher C., Feiner B., Baessler K., Christmann-Schmid C., Haya N., Brown J. (2016). Surgery for women with apical vaginal prolapse. Cochrane Database Syst. Rev..

[6] McDermott C.D., Hale D.S. (2009). Abdominal, laparoscopic, and robotic surgery for pelvic organ prolapse. Obstet. Gynecol. Clin. N. Am..

[7] Freeman R.M., Pantazis K., Thomson A., Frappell J., Bombieri L., Moran P., Slack M., Scott P., Waterfield M. (2012). A randomised controlled trial of abdominal versus laparoscopic sacrocolpopexy for the treatment of post-hysterectomy vaginal vault prolapse: LAS study. Int. Urogynecol. J..

[8] Eisenberg D., Vidovszky T.J., Lau J., Guiroy B., Rivas H. (2013). Comparison of robotic and laparoendoscopic single-site surgery systems in a suturing and knot tying task. Surg. Endosc..

[9] Lee S.R. (2016). Robotic Single-Site^®^ sacrocolpopexy: First report and technique using the Single-Site^®^ wristed needle driver. Yonsei Med. J..

[10] Lowenstein L., Matanes E., Burke Y.Z. (2017). Surgical technique for robot-assisted sacrocolpopexy performed via a sin-gle port. Urology.

[11] Liu J., Bardawil E., Zurawin R.K., Wu J., Fu H., Orejuela F., Guan X. (2018). Robotic single-site sacrocolpopexy with retroperitoneal tunneling. JSLS J. Soc. Laparoendosc. Surg..

[12] Matanes E., Lauterbach R., Mustafa-Mikhail S., Amit A., Wiener Z., Lowenstein L. (2017). Single port robotic assisted sacrocolpopexy: Our experience with the first 25 cases. Female Pelvic Med. Reconstr. Surg..

[13] Guan X., Ma Y., Gisseman J., Kleithermes C., Liu J. (2017). Robotic Single-Site sacrocolpopexy using barbed suture an-choring and peritoneal tunneling technique: Tips and tricks. J. Minim. Invasive Gynecol..

[14] Nosti P.A., Andy U.U., Kane S., White D.E., Harvie H.S., Lowenstein L., Gutman R.E. (2014). Outcomes of abdominal and minimally invasive sacrocolpopexy: A retro-spective cohort study. Female Pelvic Med. Reconstr. Surg..

[15] Unger C.A., Paraiso M.F.R., Jelovsek J.E., Barber M.D., Ridgeway B. (2014). Perioperative adverse events after minimally invasive abdominal sacrocolpopexy. Am. J. Obstet. Gynecol..

[16] Paraiso M.F.R., Jelovsek J.E., Frick A., Chen C.C.G., Barber M.D. (2011). Laparoscopic compared with robotic sacrocolpopexy for vaginal prolapse: A randomized controlled trial. Obstet. Gynecol..

[17] Pan K., Zhang Y., Wang Y., Wang Y., Xu H. (2015). A systematic review and meta-analysis of conventional laparoscopic sacrocolpopexy versus robot-assisted laparoscopic sacrocolpopexy. Int. J. Gynecol. Obstet..

[18] Matanes E., Boulus S., Lauterbach R., Amit A., Weiner Z., Lowenstein L. (2019). Robotic laparoendoscopic single-site compared with robotic multi-port sacrocolpopexy for apical compartment prolapse. Am. J. Obstet. Gynecol..

[19] Nelson R.J., Chavali J.S.S., Yerram N., Babbar P., Kaouk J.H. (2017). Current status of robotic single-port surgery. Urol. Ann..

[20] Kim J.H., Lee S.R., Lee E.S., Kim S.H., Chae H.D. (2020). Robot-assisted laparoscopic surgery for pelvic organ prolapse among peri-and post-menopausal women. J. Menopausal Med..

[21] Lee S.R., Roh A.-M., Jeong K., Kim S.H., Chae H.D., Moon H.-S. (2021). First report comparing the two types of sin-gle-incision robotic sacrocolpopexy: Single site using the da Vinci Xi or Si system and single port using the da Vinci SP system. Taiwan J. Obstet. Gynecol..

[22] Fagotti A., Bottoni C., Vizzielli G., Alletti S.G., Scambia G., Marana E., Fanfani F. (2011). Postoperative pain after conventional laparoscopy and laparoendoscopic single site surgery (LESS) for benign adnexal disease: A randomized trial. Fertil. Steril..

[23] Corrado G., Calagna G., Cutillo G., Insinga S., Mancini E., Baiocco E., Zampa A., Bufalo A., Perino A., Vizza E. (2018). The patient and observer scar assessment scale to evaluate the cosmetic outcomes of the robotic single-site hysterectomy in endometrial cancer. Int. J. Gynecol. Cancer.

